# Mutations of Residues 249 and 256 in VP2 Are Involved in the Replication and Virulence of Infectious Bursal Disease Virus

**DOI:** 10.1371/journal.pone.0070982

**Published:** 2013-07-26

**Authors:** Xiaole Qi, Lizhou Zhang, Yuming Chen, Li Gao, Guan Wu, Liting Qin, Yongqiang Wang, Xiangang Ren, Yulong Gao, Honglei Gao, Xiaomei Wang

**Affiliations:** Division of Avian Infectious Diseases, State Key Laboratory of Veterinary Biotechnology, Harbin Veterinary Research Institute, the Chinese Academy of Agricultural Sciences, Harbin, P. R. China; Institut Pasteur, France

## Abstract

Infectious bursal disease virus (IBDV) is a pathogen of worldwide significance to the poultry industry. Although the P_DE_ and P_FG_ domains of the capsid protein VP2 contribute significantly to virulence and fitness, the detailed molecular basis for the pathogenicity of IBDV is still not fully understood. Because residues 253 and 284 of VP2 are not the sole determinants of virulence, we hypothesized that other residues involved in virulence and fitness might exist in the P_DE_ and P_FG_ domains of VP2. To test this, five amino acid changes selected by sequence comparison of the P_DE_ and P_FG_ domains of VP2 were introduced individually using a reverse genetics system into the virulent strain (rGx-F9VP2). Then reverse mutations of the selected residues 249 and 256 were introduced individually into the attenuated strain (rGt). Seven modified viruses were generated and evaluated *in vitro* (CEF cells) and *in vivo* (SPF chicken). For residue 249, Q249R could elevate *in vitro* and reduce *in vivo* the replication of rGx-F9VP2 while R249Q could reduce *in vitro* and elevate *in vivo* the replication of rGt; meanwhile Q249R reduced the virulence of rGx-F9VP2 while R249Q increased the virulence of rGt, which indicated that residue 249 significantly contributed to the replication and virulence of IBDV. For residue 256, I256V could elevate *in vitro* and reduce *in vivo* the replication of rGx-F9VP2 while V256I could reduce *in vitro* but didn’t change *in vivo* the replication of rGt; although V256I didn’t increase the virulence of rGt, I256V obviously reduced the virulence of virulent IBDV. The present results demonstrate for the first time, to different extent, residues 249 and 256 of VP2 are involved in the replication efficiency and virulence of IBDV; this is not only beneficial to further understanding of pathogenic mechanism but also to the design of newly tailored vaccines against IBDV.

## Introduction

Infectious bursal disease (IBD) is a highly contagious immunosuppressive disease in chickens that has caused significant losses to the commercial poultry industry worldwide [1], [2]. Two serotypes of infectious bursal disease virus (IBDV), serotype 1 (pathogenic) and serotype 2 (non-pathogenic), have been identified. Since 1957, serotype 1 has seen the consecutive emergence of classical virulent [1], antigenic variant [3], and very virulent IBDV (vvIBDV) [4] strains that represent great challenges for the effective prevention and control of IBD.

IBDV is a member of the *Birnaviridae* family. It has a non-enveloped capsid structure containing a double-stranded RNA genome composed of two segments, A and B. Segment B encodes the VP1 protein, the viral RNA-dependent RNA polymerase [5], [6]. Segment A contains two partially overlapping open reading frames (ORFs). The smaller ORF encodes the nonstructural viral protein 5 (VP5) [7], and the larger ORF encodes a polyprotein [8]. The polyprotein is co-translationally self-cleaved to form the viral proteins VP2, VP3, and VP4 [9]. VP4 is a viral protease responsible for the self-processing of the IBDV polyprotein [10]–[12]. VP3 is a structural protein with multiple functions in the viral cycle and acts as a scaffolding protein for viral assembly [13], [14]. VP2 is the major structural protein and the only component of the icosahedral capsid [15], [16]; it is responsible mostly for virulence, cell tropism [17]–[23], and antigenic variation [24]. Two loops (P_DE_ and P_FG_) in the top domain projection of VP2 play important roles in virus infectivity in cell culture [20]–[22] and pathogenicity in chickens [22], [23]. However, the detailed molecular basis for the pathogenicity of vvIBDV is still not fully understood.

In our previous study, a vvIBDV Gx strain isolated in China was adapted to chicken embryo fibroblast (CEF) cell culture by blind passage and attenuated to form the Gt strain [25], [26]. Moreover, the virus rGx-F9VP2 with the characteristics of CEF-adaptation and moderate virulence was rescued from a Gx cDNA backbone containing two amino-acid mutations in VP2, Q253H and A284T [22]. However, vvIBDV could not be attenuated thoroughly by the combined mutations of residues 253 and 284 [22]. In the present study, an interesting gene pattern in the P_DE_ and P_FG_ domains of VP2, which might be responsible for the virulence of IBDV, was predicated by multiple sequence alignment. Then, using our previous RNA polymerase II-directed reverse genetics system [27], selected mutations were introduced into the backbone of the virulent (rGx-F9VP2) and attenuated (rGt) strains to evaluate the roles of the individual amino acids *in vitro* and *in* *vivo*.

## Materials and Methods

### Ethics Statement

All animal experiments were approved by the Harbin Veterinary Research Institute (HVRI) of the Chinese Academy of Agricultural Sciences (CAAS) and were performed in accordance with animal ethics guidelines and approved protocols. The animal Ethics Committee approval number is Heilongjiang-SYXK-2006–032.

### Viruses, Cells, and Plasmids

The vvIBDV strain Gx was previously isolated in China, identified by the INCO-China project (contract ERB IC18-CT98-0330), and attenuated to form strain Gt by blind passage [25], [26]. The virus rGt was previously rescued from the infectious clone of the Gt strain and has similar characters with the parental strain. The virus rGx-F9VP2 with the characteristics of CEF-adaptation and moderate virulence was previously rescued from a Gx cDNA backbone containing two amino-acid mutations in VP2, Q253H and A284T [22]. DF-1 cells were cultured in Dulbecco’s Modified Eagle medium (DMEM) (Invitrogen, Carlsbad, CA, USA) supplemented with 10% fetal bovine serum at 37°C in a humidified 5% CO_2_ incubator. Primary CEF cells were prepared from 10-day-old specific-pathogen-free (SPF) chicken embryos. Further propagation of the recovered virus, replication kinetics, and indirect immunofluorescence assays (IFAs) were performed in secondary CEF cells. The infectious clones pCmGtAHRT and pCmGtBHRT (containing segments A and B of rGtA) (27), the infectious clones pCGxAF9VP2HRT and pCGxBHRT (containing segments A and B of rGx-F9VP2) were constructed previously [22].

### Animals

Fourteen-day-old SPF chickens were purchased from the Experimental Animal Center of HVRI, CAAS, and housed in negative-pressure-filtered air isolators.

### Sequence Analysis of the P_DE_ and P_FG_ Domains of VP2

To find interesting amino acid residue differences in the P_DE_ and P_FG_ domains of VP2, the amino acid sequences of the P_DE_ (aa 240 to 265) and P_FG_ (aa 270 to 293) domains of VP2 of vvIBDV Gx (Accession No. AY444873), the rescued virulent strain rGx-F9VP2, and the attenuated strain Gt (Accession No. DQ403248) were compared. The alignment and phylogenetic analysis based on the nucleotide and amino acid sequences were performed using DNAStar (5.01 edition). Additionally, the three dimensional structure of VP2 from the Gx strain was built using the SWISS-MODEL workspace (http://swissmodel.expasy.org/workspace) [28]–[31].

### Site-directed Mutagenesis in the P_DE_ and P_FG_ Domains of VP2

**Figure 1:**
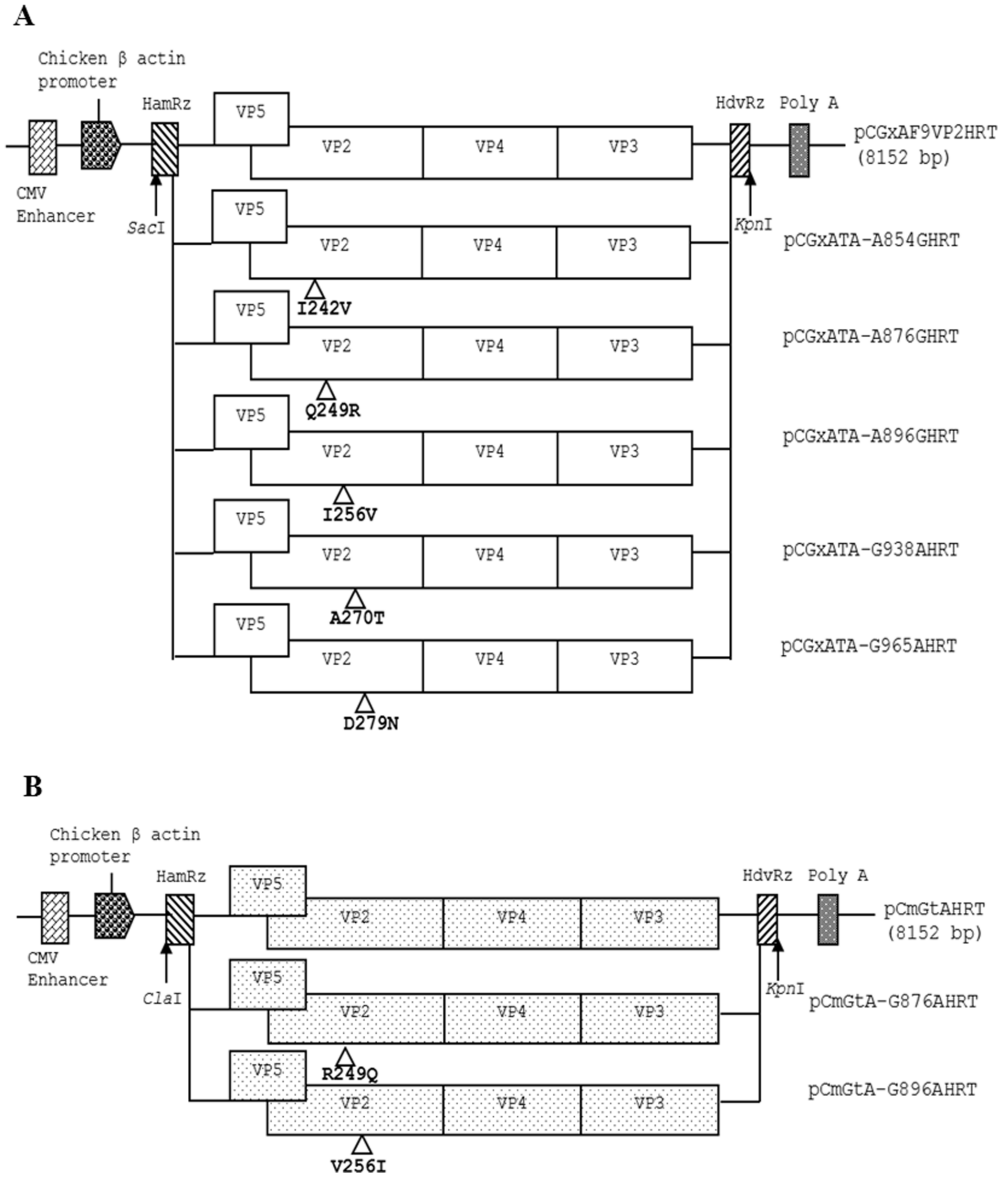
Schematic diagrams of the recombinant eukaryotic expression plasmids containing the modified cDNAs of segment A of IBDV (not drawn to scale). (A) Modified plasmids derived from pCGxAF9VP2HRT, which containing segment A of the virulent strain rGx-F9VP2 (depicted by an open box). In plasmids pCGxATA-A854GHRT, pCGxATA-A876GHRT, pCGxATA-A896GHRT, pCGxATA-G938AHRT, and pCGxATA-G965AHRT, the nucleotide substitutions A854G, A876G, A896G, G938A, and G965A resulted in the amino acid substitutions I242V, Q249R, I256V, A270T, and D279N of the VP2 protein of the virulent strain rGx-F9VP2, respectively. (B) Modified plasmids derived from pCmGtAHRT, which containing segment A of attenuated strain rGt (depicted by a box with dot). In plasmids pCmGtA-G876AHRT and pCmGtA-G896AHRT, the nucleotide substitutions G876A and G896A resulted in the amino acid substitutions R249Q and V256I in the VP2 protein of the attenuated strain rGt, respectively. The genomic cDNA sequences are preceded by a cytomegalovirus enhancer and a beta chicken actin promoter and are flanked by the cDNAs of HamRz and HdvRz. The restriction enzyme sites used for the construction of recombinant vectors are also shown.

To introduce direct mutations into the genome of the rGx-F9VP2 strain, based on the parental pCGxAF9VP2HRT plasmid, PCR for site-directed mutagenesis with specific primer pairs was developed. Five primer pairs, GxAA854GU/GxAA854GL, GxAA876GU/GxAA876GL, GxA889896U/GxA889896L, GxAG938AU/GxAG938AL, and GxA965980U/GxA965980L (Table 1), were synthesized to introduce the direct mutations A854G, A876G, A896G, G938A, and G965A (resulting in the amino acid mutations I242V, Q249R, I256V, A270T, and D279N in VP2) into segment A of the rGx-F9VP2 strain, respectively. PCR amplification was performed for 18 cycles of denaturation at 98°C for 30 s, and annealing and extension at 72°C for 8 min. The PCR was directed by PrimeSTAR™ HS DNA Polymerase (Takara Biotechnology Co., Ltd., Dalian, China). The PCR products were digested with *Dpn*I (NEB, Ipswich, England) for 1 h at 37°C to remove DNA of the methylated parental plasmid. The digested PCR products were used to transform competent cells to amplify the resulting plasmids, pCGxATA-A854GHRT, pCGxATA-A876GHRT, pCGxATA-A896GHRT, pCGxATA-G938AHRT, and pCGxATA-G965AHRT (Figure 1).

**Table 1:** Primers for genome cloning and mutagenesis of IBDV.^a^

Name	Sequence	Orientation	Position (nt)
GxAA854GU	AGTCTCAGCgTCGGGGGAGA	sense	A: 845∼864
GxAA854GL	TCTCCCCCGAcGCTGAGACT	antisense	A: 845–864
GxAA876GU	TCGTGTTTCgAACAAGCGTC	sense	A: 867∼886
GxAA876GL	GACGCTTGTTcGAAACACGA	antisense	A: 867∼886
GxA889896U	TCCATGGCCTTgTACTGGGTGC	sense	A: 885∼906
GxA889896L	GCACCCAGTAcAAGGCCATGGA	antisense	A: 885∼906
GxAG938AU	TGATGGGACTaCGGTAATCACC	sense	A: 928∼949
GxAG938AL	GGTGATTACCGtAGTCCCATCA	antisense	A: 928∼949
GxA965980U	TGTGGCCGCAaACAATGGGCTAACGACCGGCACTGA	sense	A: 955∼990
GxA965980L	TCAGTGCCGGTCGTTAGCCCATTGTtTGCGGCCACA	antisense	A: 955∼990
GtAG876AU	TCGTGTTTCgAACAAGCGTC	sense	A: 867∼886
GtAG876AL	GACGCTTGTTcGAAACACGA	antisense	A: 867∼886
GtAG896AU	TCCAAGGCCTTgTACTGGGTGC	sense	A: 885∼906
GtAG896AL	GCACCCAGTAcAAGGCCTTGGA	antisense	A: 885∼906
GxAU	G*GAATTC*GGATACGATCGGTCTGAC	sense	A: 1∼18
GxA1477L	AGGTAGCCCATGTCTGGT	antisense	A: 1460∼1477
B3P	ACTACCCACTCCTGAACAAA	sense	B:2009∼2028
B37	GC*TCTAGA*GGGGGCCCCCGCAGGCGAAGGCCGGGGAT	antisense	B: 2799∼2827

a The positions where the primers GtAG876AU, GtAG876AL, GtAG896AU, and GtAG896AL bind are in accordance with the published sequence of IBDV strain Gt (GenBank assession nos: DQ403248). The positions where other primers bind are in accordance with the published sequence of strain Gx (GenBank assession nos: AY444873 and AY705393). The introduced mutations are lower-case characters. Orientation and position of the primers are shown.

Similarly, based on the sequences of segment A in the parental plasmid pCmGtAHRT, the primer pairs GtAG876AU/GtAG876AL or GtAG896AU/GtAG896AL (Table 1) were used to introduce the nucleotide mutations G876A and G896A (resulting in the amino acid mutations R249Q and V256I in VP2) into segment A of rGt to obtain the two modified plasmids, pCmGtA-G876AHRT and pCmGtA-G896AHRT (Figure 1), respectively. All of the modified plasmids were sequenced at least three times for confirmation.

### Rescue and Identification of Modified IBDV

To rescue virus using the reverse genetics system directed by RNA polymerase II [27], purified plasmids with the rGx-F9VP2 backbone (pCGxATA-A854GHRT, pCGxATA-A876GHRT, pCGxATA-A896GHRT, pCGxATA-G938AHRT, and pCGxATA-G965AHRT) were each co-transfected with pCGxBHRT into DF-1 cells, respectively. Purified plasmids with the rGt backbone (pCmGtA-G876AHRT and pCmGtA-G896AHRT) were also co-transfected with pCmGtBHRT, respectively. At 72 h post-transfection, after freezing and thawing for three times, the rescued viruses were harvested from the supernatants by centrifugation at 3000×g for 10 min at 4°C and used to infect secondary CEF cells. The modified viruses were blind passaged six times in CEF cells prior to subsequent experiments. To characterize the modified viruses, IFA with anti-VP2 mAb, an electron microscopy assay, and RT-PCR using the primer pairs GxAU/GxA1477L and B3P/B37 (Table 1) were performed as described previously [27].

### Replication Kinetics of Modified IBDV in CEF Cells

The replication kinetics curves were obtained to assess the replication abilities of the modified IBDV and the control rGx-F9VP2 or rGt. Confluent secondary CEF cells in 60 mm culture plates (approximately 10^6^ cells/plate) were infected with each virus strain at a 50% cell culture infective dose (TCID_50_) of 10^4^, and subsequently harvested at 12 h intervals. The titer of the infectious viral progeny was determined as TCID_50_ per milliliter using the Reed-Muench formula. The mean values and standard deviations of the data obtained from three independent experiments were calculated.

### Characterization of Modified IBDV in Chickens

Two animal experiments were designed in order to evaluate the virulence of the modified viruses with the rGx-F9VP2 (i) and the rGt backbone (ii). SPF chickens housed separately in negative-pressure isolators were infected with viruses or DMEM intranasally and by eye drops.

In experiment i, fourteen-day-old chickens were randomly assigned to seven groups with 21 chickens per group. The first and second groups were infected with 10^5.8^ TCID_50_ of the modified IBDV rGxHT-249. The third and fourth groups were infected with 10^5.8^ TCID_50_ of the modified IBDV rGxHT-256. The fifth and sixth groups were infected with 10^5.8^ TCID_50_ of the control rGx-F9VP2. The seventh group received 200 µl of DMEM without virus as a negative control. The second, fourth, sixth groups were only used to evaluate the mortality of each virus.

In experiment ii, fourteen-day-old chickens were randomly assigned to four groups with 21 chickens per group. The first, second, and third groups were infected with 10^5.8^ TCID_50_ of the modified IBDV rGt-249, rGt-249, and rGt, respectively. The fourth group received 200 µl of DMEM without virus as a negative control.

Chickens were observed daily for clinical symptoms. At 1, 2, 3, 5, 7, 10, and 14 days post-inoculation (d p.i.), three chickens were randomly selected from each group (except the second, fourth, and sixth groups in experiment i), euthanized for necropsy, and examined for signs of pathological changes. The bursa and body weights of all chickens were determined, and the bursa:body-weight index (BBIX) at 1, 2, 3, 5, 7, 10 and 14 d p.i. was calculated with standard deviation (BBIX = (bursa:body-weight ratios)/(bursa:body-weight ratios in the negative group)). The mean values and standard deviations of the data obtained from three independent chicken samples were calculated. Bursae with a BBIX less than 0.70 were considered atrophied [32]. Each bursa was then cut into 2 parts, one piece for histopathological assay and the other for detecting viral gene.

### Replication Kinetics of the Modified IBDV *in vivo*

To evaluate the replication of the modified virus *in vivo*, at 1, 2, and 3 days post-infection, viral RNA obtained from the bursae of chickens inoculated with the rescued viruses and the DMEM control were quantified by real-time RT-PCR as described previously [33]. The mean values and standard deviations of the data obtained from three independent chicken samples were calculated.

### Histopathology

The bursae from each group isolated at different days were fixed immediately after necropsy in 10% neutrally buffered formalin and were stained with hematoxylin and eosin for histopathological examination as described previously [22]. The severity of bursal follicular necrosis was recorded using the average histopathological bursa lesion score (HBLS) system as described previously [34].

### Identification of the Modified IBDV from the Bursae

To confirm the identity of the modified virus and investigate whether changes in the nucleotide sequence occurred during challenge, the viral RNAs obtained from the chicken bursae were amplified by RT-PCR and then sequenced as mentioned above.

### Statistical Analyses

One-way ANOVA was employed to evaluate the significance of differences among different groups. P<0.05 were considered significant differences.

## Results

### Sequence Analysis of the P_DE_ and P_FG_ Domain of VP2

**Figure 2:**
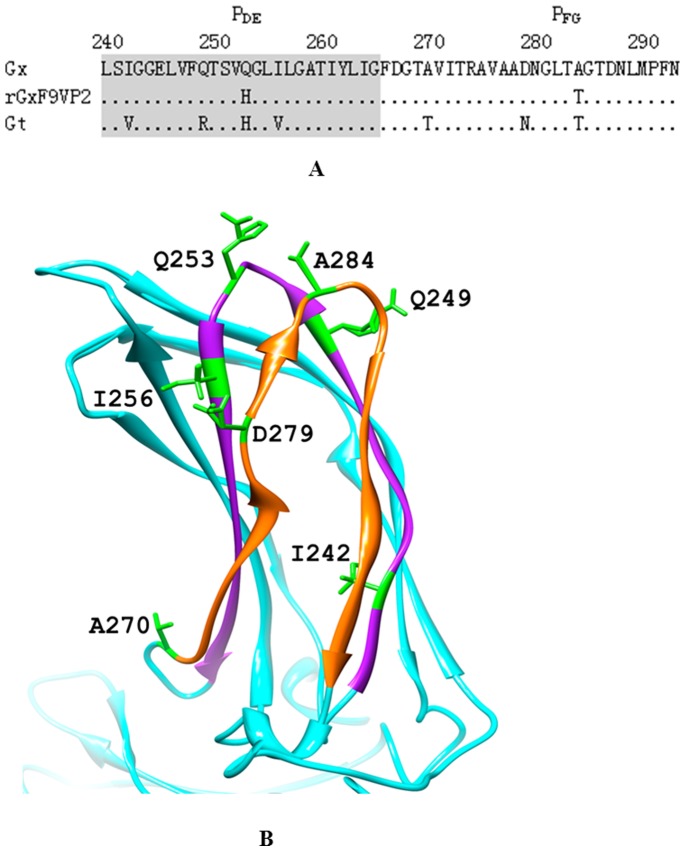
Comparison of the amino acid sequences of the P_DE_ and P_FG_ domain of IBDV VP2. (A) Amino acid sequences of the P_DE_ (aa 240 to 265) and P_FG_ (aa 270 to 293) domains of VP2 of vvIBDV Gx (GenBank Accession No. AY444873), the rescued virulent strain rGx-F9VP2, and the attenuated strain Gt (GenBank Accession No. DQ403248) were compared. The P_DE_ and P_FG_ domains are marked, respectively. (B) The three-dimensional structure of VP2 of the Gx strain was built by the SWISS-MODEL workspace (http://swissmodel.expasy.org/workspace) and depicted using UCSF Chimera 1.6.1 (http://www.cgl.ucsf.edu/chimera); only the projection domain is shown here. P_DE_ (purple) and P_FG_ (orange) domains are highlighted with different color. The different residue sites of the P_DE_ and P_FG_ domains among these strains are highlighted with green color: I242, Q249, Q253, I256, A270, D279, A284.

Sequence alignment showed that compared with Gx, rGx-F9VP2 had two mutations, one at loop P_DE_ (Q253H) and one at loop P_FG_ (A284T); Gt strain, in addition to mutations at residues 253 and 284, had another five mutations of I242V (strand P_D_), Q249R (strand P_D_), I256V (strand P_E_), A270T (strand P_F_), and D279N (strand P_F_) in domains P_DE_ and P_FG_ of VP2 (Figure 2). The three dimensional structure of VP2 from the Gx strain showed that residues 249 and 256 are both located in the P_DE_ domain at the tip of the VP2 spikes; they also surround residue 253 and are next to residue 284 (Figure 2).

### Rescue of Modified IBDV

To test the function of residues 242, 249, 256, 270, and 279 in the P_DE_ and P_FG_ domains of VP2, a set of mutated infectious clones with the backbone of the virulent strain (rGx-F9VP2) were constructed, respectively. Through identification with IFA, electron microscopy assay, and sequencing (data not shown), the modified IBDV were rescued in cells co-transfected with plasmids containing the rGx-F9VP2 backbone (pCGxATA-A854GHRT, pCGxATA-A876GHRT, pCGxATA-A896GHRT, pCGxATA-G938AHRT, and pCGxATA-G965AHRT) and pCGxBHRT; the rescued viruses were named rGxHT-242, rGxHT-249, rGxHT-256, rGxHT-270, and rGxHT-279, which contained mutations in the P_DE_ domain (I242V, Q249R, and I256V) and the P_FG_ domain (A270T and D279N) of VP2 in the backbone of the virulent strain rGx-F9VP2, respectively. Through co-transfection with the plasmids of the rGt backbone (pCmGtA-G876AHRT and pCmGtA-G896AHRT) and pCmGtBHRT, the mutated viruses rGt-249 and rGt-256 were rescued, which contained the mutations R249Q or V256I in the P_DE_ domain of VP2 in the backbone of the attenuated strain rGt, respectively.

### Replication of the Modified IBDV *in vitro* and *in vivo*

**Figure 3:**
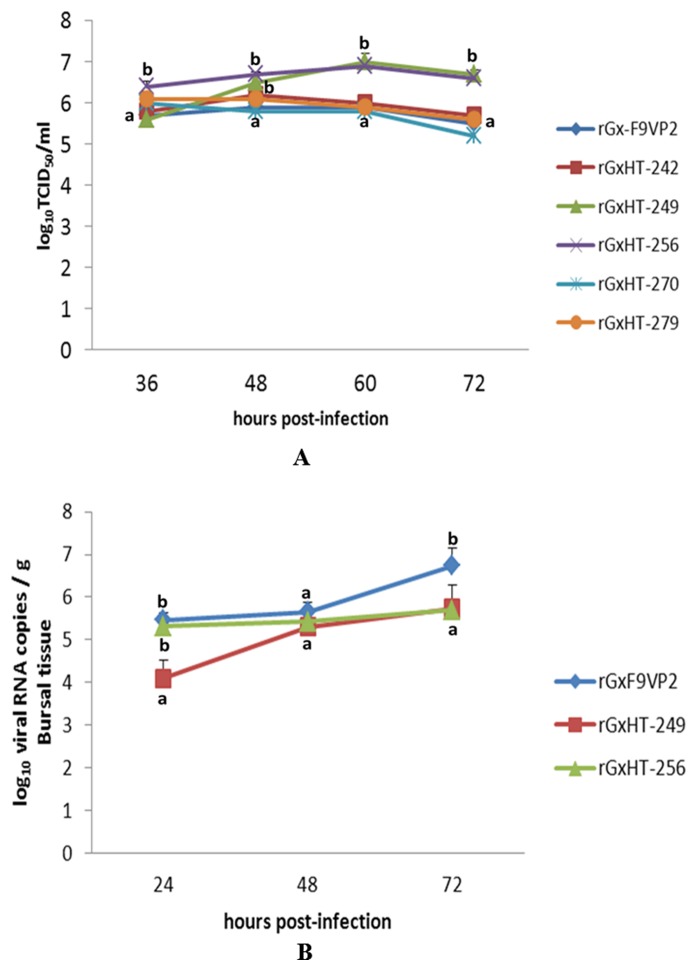
Replication kinetics curves of modified IBDV strains based on the backbone of virulent strain rGx-F9VP2. (A) Secondary CEF cells (approximately 10^6^ cells) were infected with the modified viruses based on rGx-F9VP2 backbone at 10^4^ TCID_50_. The viral titers harvested at different time intervals were calculated and expressed as TCID_50_ per milliliter. (B) Fourteen-day-old SPF chickens were infected with 10^5.8^ TCID_50_ of the modified virus based on the rGx-F9VP2 backbone via eye and intranasal routes. The BF was collected and homogenized at the indicated time points. Subsequently, viral RNA loads were quantified using real-time RT-PCR. Data were the mean titer (log of viral RNA copies) per g of tissue. Average titers and standard deviations (error bars) from three independent samples are shown. Treatments sharing different lowercase letter differ significantly at a confidence level (P<0.05).

All of the modified IBDV could induce typical cytopathogenic effects (CPEs) in CEF cells; these CPEs became more significant after blind passage. To investigate the difference in the replication efficiency of IBDV in detail, the replication kinetics curve for each virus in CEF cells was depicted (Figure 3A). Compared with the parental strain of rGx-F9VP2, rGxHT-249 and rGxHT-256 replicated more quickly in CEF cells. At 60 hours post infection (h p.i.), rGxHT-249 and rGxHT-256 reached the highest titers of 10^7^ and 10^6.9^ TCID_50_/ml, respectively, which were over 10 times higher than rGx-F9VP2 (10^5.9^ TCID_50_/ml) (P<0.05). Meanwhile, rGxHT-242, rGxHT-270, and rGxHT-279 showed similar curves to rGx-F9VP2 (P>0.05). *In vivo*, the viral replication properties in the bursa as measured by real-time RT-PCR (Figure 3B) showed that compared with rGx-F9VP2, the replication of rGxHT-249 and rGxHT-256 was delayed in the bursa. At 72 h p.i., rGxHT-249 and rGxHT-256 reached titers of 10^5.74^ and 10^5.7^ viral RNA copies/g, respectively, which were over 10 times lower than rGx-F9VP2 (10^6.74^ copies/g) (P<0.05).

**Figure 4:**
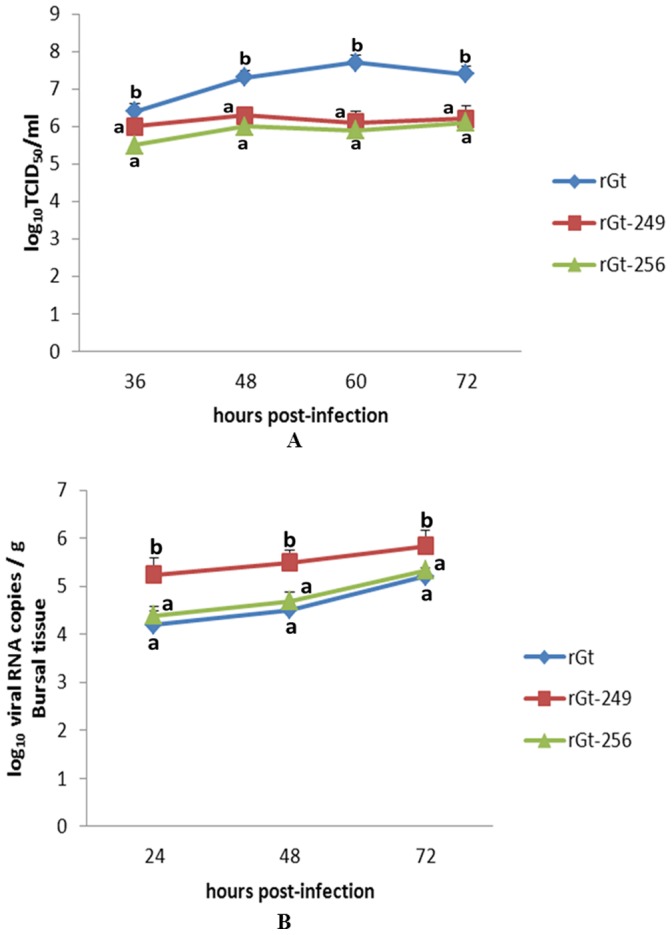
Replication kinetics curves of modified IBDV strains based on the backbone of attenuated strain rGt in secondary CEF cell (A) and in bursa (B). Experiments were performed as described for Fig. 3. Average titers and standard deviations (error bars) from three independent samples are shown. Treatments sharing different lowercase letter differ significantly at a confidence level (P<0.05).

Compared with rGt, rGt-249 and rGt-256 replicated more slowly in CEF cells (Figure 4A). At 60 h p.i., rGt-249 and rGt-256 reached titers of 10^6.1^ and 10^5.9^ TCID_50_/ml, respectively, which were 40 and 63 times lower than rGt (10^7.7^ TCID_50_/ml) (P<0.05). *In vivo*, rGt-249 had higher titer in bursa than rGt (Fig. 4B). At 24 and 48 h p.i., rGt-249 reached titers of 10^5.24^ and 10^5.49^ viral RNA copies/g, which were about 10 times higher than rGt (P<0.05). Meanwhile, rGt-256 showed similar curve to rGt in bursa (P>0.05) (Figure 4B).

### Virulence of the Modified IBDV *in vivo*

**Figure 5:**
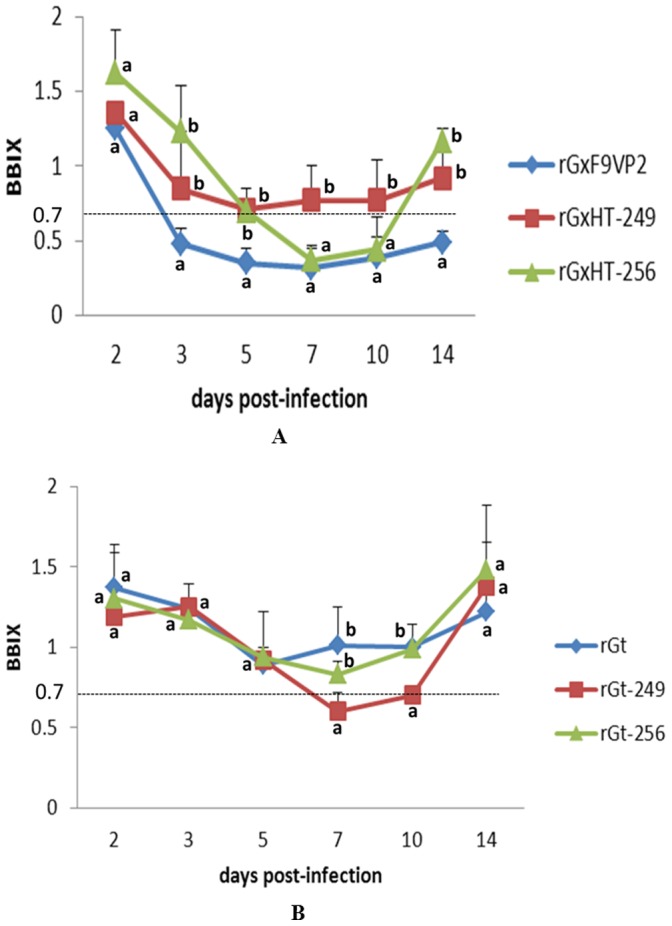
Kinetics curves of bursa:body-weight index (BBIX) of SPF chickens infected with the modified virus based on the backbone of virulent strain rGx-F9VP2 (A) and attenuated strain rGt (B). The bursa and body weights of all chickens were determined, and BBIX at different days post-infection was calculated with standard deviation (BBIX = (bursa:body-weight ratios)/(bursa:body-weight ratios in the negative group)). Bursa with a BBIX of less than 0.70 were considered atrophied. Average titers and standard deviations (error bars) from three independent samples are shown. Treatments sharing different lowercase letter differ significantly at a confidence level (P<0.05).

In experiment i, the *in vivo* virulence of the modified IBDV rGxHT-249 and rGxHT-256 was investigated in fourteen-day-old SPF chickens. Throughout the experimental period, neither death nor typical clinical symptoms of IBD were observed in any of the groups. However, bursa atrophy with obvious differences were observed between chickens infected with rGx-F9VP2 and those infected with rGxHT-249 and rGxHT-256. To research the process of bursa atrophy induced by different viruses, BBIX at different d p.i. were calculated (Figure 5A). The BBIX of the rGx-F9VP2 group was below 0.7 from 3 d p.i. to 14 d p.i., while in the rGxHT-256 group, a BBIX below 0.7 was only observed from 7 d p.i. to 10 d p.i. and then recovered to 1.17 at 14 d p.i. More interestingly, the BBIX of the rGxHT-249 group was always higher than 0.7 post infection.

In experiment ii, the *in vivo* virulence of the modified IBDV rGt-249 and rGt-256 was evaluated. No clinical symptoms of IBD were observed in each group. The BBIX of the rGt-256 and rGt groups were always higher than 0.7 post infection. However, in rGt-249 group, BBIX of 0.6 was observed at 7 d p.i. and it was still low (BBIX = 0.7) at 10 d p.i. (Figure 5B).

### Histopathological Examination of the Bursa

**Figure 6:**
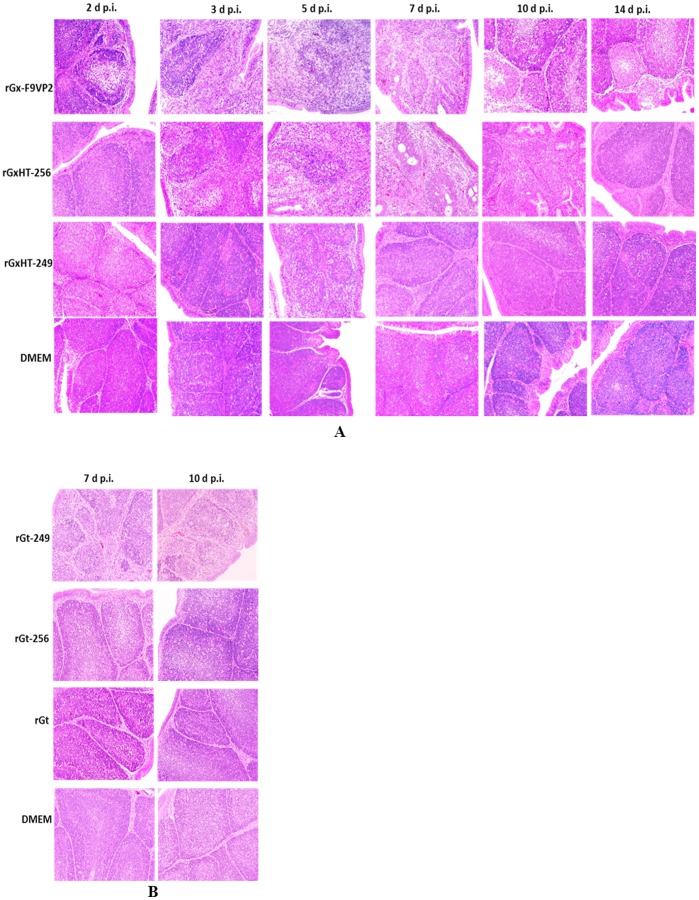
Histopathological appearance of bursal sections (hematoxylin and eosin) derived from groups of chickens infected with the modified viruses based on the backbone of virulent strain rGx-F9VP2 (A) and attenuated strain rGt (B) at different days post-infection (X 200). (A) The virulent strain rGx-F9VP2 induced moderate and persistent histopathological bursal lesions including middling lymphocytic deletion and necrosis, regional atrophy, and fibrosis of the follicle. In the rGxHT-256 group, no pathological lesions were observed at 2 d p.i.; slight lymphocytic deletion and necrosis was observed at 3 and 5 d p.i.; moderate lesions including atrophy and mild vacuolation of the follicle were observed at 7 d p.i.; and then the bursa began to recover from 10 d p.i. and returned to normal at 14 d p.i. The rGxHT-249 group presented no pathological lesions other than very slight lymphocytic deletions at 5 d p.i. There were no microscopic lesions of the bursa in the control chickens infected with DMEM. (B) The atrophy of the follicle, lymphocytic deletion, and connective tissue hyperplasia appeared in the bursa of rGt-249 group at 7 and 10 d p.i. No bursal lesions were observed in rGt-256, rGt, and DMEM group.

The histopathological changes of the infected bursae at different d p.i. were presented (Figure 6). In experiment i (Figure 6A), the virulent strain rGx-F9VP2 induced moderate and persistent histopathological bursal lesions including middling lymphocytic deletion and necrosis, regional atrophy, and fibrosis of the follicle. In contrast, relative slighter pathological changes to the bursae were induced by rGxHT-256, and the changes were delayed. In the rGxHT-256 group, no pathological lesions were observed at 2 d p.i.; slight lymphocytic deletion and necrosis was observed at 3 and 5 d p.i. (average HBLS was below 3); relative severer lesions including atrophy and mild vacuolation of the follicle with an average HBLS of 4 were observed at 7 d p.i.; and then the bursa began to recover from 10 d p.i. and returned to normal at 14 d p.i. Compared with rGx-F9VP2 and rGxHT-256, the rGxHT-249 group presented no pathological lesions other than very slight lymphocytic deletions with an average HBLS of 2 at 5 d p.i. In addition, there were no microscopic lesions of the bursa in the control chickens infected with DMEM (Figure 6A). In experiment ii, the atrophy of the follicle, lymphocytic deletion, and connective tissue hyperplasia appeared in the bursa of rGt-249 group at 7 and 10 d p.i. (Figure 6B). No obvious bursal lesions were observed in rGt-256, rGt, and DMEM group (Figure 6B).

### Confirmation of Genetic Stability of the Modified Virus

RT-PCR and sequencing was performed to confirm the presence of modified viruses in the bursae. The sequence analysis showed that modified viruses replicated with high fidelity in the bursae of chickens and that their gene sequences did not revert to that of the parental strains. In addition, these modified viruses were shown to be genetically stable after 10 passages in the secondary CEF cells.

## Discussion

Similar to other RNA viruses, IBDV is genetically prone to mutation. During the past 50 years of evolution, the classic [1], antigenic variant [3] and very virulent IBDV (vvIBDV) strains [4] have emerged in succession, representing new challenges for the effective prevention and control of IBD. There is a potential risk that IBDV might further evolve to a more virulent state because of new environments. At the same time, passaging pathogenic strains of the virus in cell culture has been used to attenuate IBDV [26], [35], but this is a time consuming and laborious process with uncertain results for vaccine development. Recently, reverse genetics has made it possible to develop rapidly tailored vaccines suitable against the popular strain [36]. However, there remains an information bottleneck regarding gene function, especially the precise molecular determinants of virulence and replication efficiency of IBDV that are not fully understood.

As the only component of the icosahedral capsid, VP2 is folded into three distinct domains, designated base (B), shell (S), and projection (P) [15], [37], [38]. The domains B and S are relatively well conserved, but domain P is more variable. The tower-like P domain contains four loops, P_BC_, P_HI_, P_DE_, and P_FG_. Furthermore, it has been reported that the P_DE_ and P_FG_ domains are responsible for cell-tropism and virulence [18], [20]–[23], while the P_BC_ and P_HI_ domains are involved in antigenicity mutations of IBDV [24]. Previously, we discovered that residue 253 and 284, located in loops P_DE_ and P_FG_ at the tip of the VP2 spike, were the most involved in virulence and cell-tropism in Gx strain of IBDV [22], which enriches previous reports [17], [18], [21], [23]. The vvIBDV Gx strain was adapted to CEF cell culture and attenuated to form the rGx-F9VP2 strain by the combined mutations of Q253H and A284T. However, we also found that rGx-F9VP2 could not be attenuated thoroughly, as it could still induce histopathological bursal lesions [22]. This suggested that in addition to the two known residues, it would be interesting to study other residues in the P_DE_ and P_FG_ domains of VP2 to see if they might be involved in the virulence of IBDV.

To verify the hypothesis, aimed at five amino acid residues differences in the P_DE_ and P_FG_ domains of VP2 between virulent strain rGx-F9VP2 and attenuated strain rGt, I242V (strand P_D_), Q249R (strand P_D_), I256V (strand P_E_), A270T (strand P_F_), and D279N (strand P_F_) mutations were introduced into the backbone of rGx-F9VP2, the corresponding mutated viruses (rGxHT-242, rGxHT-249, rGxHT-256, rGxHT-270, and rGxHT-279) were then rescued, respectively. The replication characteristics of the mutated viruses were firstly evaluated in CEF cells. Among five mutations, only rGxHT-249 and rGxHT-256 replicated more quickly in CEF cells with over 10 times than the parental strain of rGx-F9VP2. In addition, in the bursa of infected chickens, the replication of rGxHT-249 and rGxHT-256 was delayed, with the viral load being over 10 times lower than that of rGx-F9VP2. These data showed that residue mutation Q249R or I256V of VP2 could promote the propagation in CEF cells and reduce the replication in bursa of the virulent IBDV strain rGx-F9VP2.

To further research whether the reverse mutations of residues 249 or 256 could influence replication efficiency of IBDV, the mutation R249Q or V256I was introduced into the backbone of the rescued attenuated strain rGt. The replication kinetics curves showed that the additional mutations R249Q and V256I could reduce the titers of rGt in CEF cells 40 and 63 times, respectively. In bursa, R249Q could significantly increase the replication of rGt while V256I couldn’t. Usually, to some extent, the replication efficiency *in vitro* and *in vivo* of IBDV negatively correlated each other. For residue 249 of VP2, Q249R could elevate *in vitro* and reduce *in vivo* the replication of the virulent IBDV rGx-F9VP2. Inversely, R249Q could reduce *in vitro* and elevate *in vivo* the replication of attenuated IBDV rGt. The results observed from different backbones *in vitro* and *in vivo* verified that residues 249 of VP2 were obviously involved in influencing the replication efficiency of IBDV.

About residue 256 of VP2, to be mentioned, it’s a little different than expected that V256I didn’t influence the replication of rGt in bursa. The function of residue 256 maybe more depend on the surroundings of the genome backbone than other residues. However, it’s convincing that V256I significantly reduce the replication of rGt in CEF cells, and the reverse mutation I256V could elevate *in vitro* and reduce *in vivo* the replication of the virulent IBDV rGx-F9VP2. These data supported that residue 256 of VP2 were also partly involved in influencing the replication efficiency of IBDV. For IBDV, different replication characters usually predict different virulence. For example, compared with virulent strains, attenuated strains usually replicate slower in bursa but faster *in vitro*. Le Nouen et al. have speculated that the conserved amino acids in vvIBDVs and the 88180 strain including residue 256 might be involved in virulence [40]. However, no further experiments were performed to verify it.

To verify whether residues 249 and 256 of VP2 contribute to the virulence of IBDV, animal experiments using SPF chickens were performed. As rGx-F9VP2, rGxHT-249 and rGxHT-256 groups presented neither death nor typical clinical symptoms. For sub-clinical disease, the degree of damage to the bursa and reductions in the BBIX are good index of virulence and have been used in many studies [5], [17]–[19], [22], [41]–[43]. Based on the BBIX value, bursa atrophy with obvious differences was observed. Compared to rGx-F9VP2, which exhibits severe bursa atrophy, only transient bursa atrophy was observed in the rGxHT-256 group. The results were further confirmed by the histopathogical examination of the bursa. However, as attenuated rGt, the BBIX of the rGt-256 group were always higher than 0.7 post infection. Usually, there was a positive correlation between the replication efficiency *in vivo* and the virulence of IBDV. As mentioned above, the replication efficiency in bursa of attenuated rGt didn’t be altered by mutation V256I so that its virulence didn’t be elevated. Although V256I didn’t increase the virulence of attenuated strain, I256V significantly reduced the virulence of virulent IBDV, which indicated residue 256 of VP2 partly involved in the virulence of IBDV.

For the rGxHT-249 group, the additional residue mutation Q249R reduced the virulence of the rGx-F9VP2 strain even more, and no statistically significant bursa atrophy was found. Meanwhile, the reverse mutation R249Q obviously increased the virulence of rGt, obvious bursal lesions were found at 7 and 10 d p.i. The results observed from different backbones showed that residue 249 of VP2 significantly contributed to the virulence of IBDV.

Taken together, to different extent, both residues 249 (strand P_D_) and 256 (strand P_E_) of VP2 contribute to the virulence of IBDV. Notably, the rGxHT-249 group presented no pathological lesions other than very slight lymphocytic deletion at 5 d p.i. Our previous report showed that compared to the Gx strain with a mortality of over 60%, vvIBDV was markedly attenuated by Q253H/A284T, as the modified virus rGx-F9VP2 caused no mortality [22]. This study presented that rGx-F9VP2, but not rGxHT-249, induced bursa atrophy, which might show that the tri-mutation Q249R/Q253H/A284T of VP2 could attenuate vvIBDV more thoroughly.

How these residues mutations may influence the replication and virulence of IBDV is unclear. Both residues 249 and 256 are located in the P_DE_ domain at the tip of the VP2 spike; the position of both residues are close to or belong to minor hydrophilic peak A (aa 247–254 of VP2), which was suspected to have strong antigenic significance; they also surround residue 253 and lie next to residue 284, which determine the cell-tropism of IBDV. To the best of our knowledge, reports regarding residues 249 and 256 of VP2 of IBDV are few. Recently, the characteristics of residues 222, 286, 318, and 249 were used to perform a phylogenetic analysis of IBDV [39], which suggested that residue 249 might contribute to the differences between various strains. To be mentioned, the amino acid residue at position 249 is changed from an uncharged Q to a positively charged R. Molecular charge is usually involved in the assembly and stability of the virus. It’s well known that wild IBDV replicates well in the target B-lymphoid cells of bursa but doesn’t adapt to CEF cells, while attenuated strain can replicate in both cells but the replication efficiency in B-lymphoid cells decreases. The cellular mechanism being hijacked to facilitate its entry and replication is still largely unknown. It’s speculated that some mutations lead to a conformational change in VP2, resulting in gaining the recognition of the general IBDV receptor presented on a wide range of cells including CEF, and leaving the recognition of the special B-lymphoid IBDV receptor intact to some extent [17]. Recently, researchers suspected that birnaviruses including IBDV might need to interact with two different cell receptors during entry, one for attachment located at the top of the spike of VP2 which may confer tissue tropism and modulate virulence, and another for internalization located at the conserved groove of VP2 [44]. Residues 249 and 256 in themselves unlikely determine cell-tropism but are likely involved in interactions that allow other amino acids to contribute to the replication and virulence of IBDV. The detailed mechanism controlling the function of residues 249 and 256 of VP2 deserves further study.

In conclusion, for the first time, this study demonstrates that residues 249 and 256 of VP2 are involved in the replication efficiency and virulence of IBDV. Moreover, introducing the additional mutation Q249R to the basal Q253H/A284T mutation of VP2 could attenuate IBDV more thoroughly. These findings are not only beneficial to our further understanding of the mechanism of viral virulence, but also significant to the design of new tailored vaccines for IBDV.
